# Will better evidence on clinical utility bring about greater use of (genetic) tests?

**DOI:** 10.1038/s41525-021-00187-8

**Published:** 2021-03-04

**Authors:** Chris Hyde

**Affiliations:** Exeter Test Group, Institute of Health Research, College of Medicine and Health, University of Exeter, Exeter, EX1 2LU UK

**Keywords:** Epidemiology, Predictive markers

## Abstract

Greater clarity on the nature of clinical utility is desirable. Of itself it may not bring about greater use of tests, including WGS (whole-genome sequencing), not least because clinical utility studies when performed may not confirm predicted changes in patient outcome. The notion that single “pivotal” clinical utility studies will achieve uptake needs to be questioned and that the evidence base for tests is likely to rely on patchworks of imperfect evidence embraced.

Evaluation of tests is difficult. I have seen just how difficult in the work I have done contributing to decision making on NICE’s Diagnostic Assessment Committee and the National Screening Committee in the UK over the last decade. “Clinical utility of genomic sequencing: a measurement toolkit”^1^ is a helpful contribution to this challenging area. It builds on existing frameworks, particularly that proposed by Fryback and Thornbury^2^ and is particularly useful in offering WGS-specific examples of clinical utility studies. The emphasis on comparison is welcome, and is an aspect of test evaluation which has been strangely lacking^3^. Clear demonstration of how a new test will lead to improvement relative to existing test strategies, must be the foundation of any decisions on whether a test is recommended for wide use, irrespective of whether the impact falls under the category of analytical or clinical validity or clinical utility.

Although the checklist does represent a useful contribution, it is one which needs to be built on further:

- The current perspective is wholly clinical. Utility in particular will vary with perspective, and account must be taken of this. Patients and carers have legitimate views as do policymakers and funders and the toolkit should reflect these and other stakeholder views as much as the medical community. “*Clinical* utility” is a misnomer; its intent is to reflect how a new test will impact on patient outcome and judgement on what constitutes this must be contributed to widely.
- The toolkit largely focuses on what should be measured. Equal emphasis needs to be devoted to how, with more space devoted to study design and the relative strengths and weaknesses of different methods of data collection. The toolkit would benefit from greater scrutiny of its examples commenting on the role of chance, bias and confounding on the interpretation of the results. Accumulation of data in a way which does not maximise its validity wastes everyone’s time.
- The authors acknowledge their lack of health economic expertise, so advice on capturing the cost-effectiveness of WGS should be enhanced, particularly because achieving diagnosis more efficiently is often central to the WGS case. There is an absence of any mention of modelling which has a critical role in evidence linkage, both for the purposes of establishing effectiveness and cost-effectiveness^4,5^.
- Evaluators are likely to be bewildered by the range of outcomes they are being asked to evidence, so the advice ideally needs to assist with prioritisation or strategies to help build a portfolio of evidence which will convince funders and society that introduction of WGS does more good than harm (an issue which is barely mentioned in the current toolkit). As for test evaluation, generally, emphasising the key claims central to any value proposition and pre-eminent threats to safety should be the first concerns.
- Finally, advice is needed on the synthesis of evidence as much as its initial collection. The toolkit cites meta-analyses of evidence, which illustrate that improvements to secondary research can be made^6^. This is particularly true for the task of summarising evidence from the disparate aspects clinical utility. However, to truly create a convincing portfolio of evidence, the ambition should be to incorporate key evidence relating to analytical and clinical validity too.

Even if the clinical utility toolkit is optimised, will this prove sufficient in getting genetic tests into practice? This seems unlikely as ideas on the evidence requirements for tests in general, and genetic tests in particular have been available for some time^7^. So other issues need to be examined to see whether there are other important rate-limiting steps to the up-take of WGS. Funding, in my view, is the major constraint. While research funders have been quick to back investment in the technology they have been cautious in supporting its evaluation, which, as the toolkit highlights, is complex requiring dedicated and experienced evaluation teams. Related to this, we also have to consider whether geneticists advocating the technologies really believe they should have to go beyond analytical validity. As a colleague recently put it when we were discussing a potential trial to demonstrate clinical utility, “Why should we have to do more than other tests?” And we should acknowledge that there is some truth in this sentiment as historically new tests have had to do little more than demonstrate their laboratory performance. We also need to consider that clinical utility studies, when performed, are not always favourable, and that however persuasive the rationale, the hoped-for benefits are not realised in practice. The toolkit contains at least one example where the benefits were less than that might have been hoped for when they were examined rigorously^8^.

A last word of caution is that decisions on tests will probably always rely on patchworks of imperfect evidence. The notion, which underlies many evaluation frameworks that a “pivotal” clinical utility study will at some stage firmly establish the effectiveness of a test needs to be questioned. Making more of what we already have by better collating and synthesising evidence portfolios may be as fruitful as holding out for the mirage of the perfect study which will single-handedly tip the balance of getting a new WGS into practice. Targeted augmentation of a body of evidence to demonstrate specific missing aspects of a value proposition may be more valuable than pursuing a clinical utility study for its own sake.

## Reporting summary

Further information on research design is available in the Nature Research Reporting Summary linked to this article.

## Supplementary information

Reporting Summary
